# Competency-based teacher training: A systematic revision of a proven programme in medical didactics

**DOI:** 10.3205/zma001121

**Published:** 2017-10-16

**Authors:** Jan Griewatz, Melanie Simon, Maria Lammerding-Koeppel

**Affiliations:** 1 University of Tuebingen, Competence Centre for University Teaching in Medicine Baden-Wuerttemberg, Tübingen, Germany; 2 RWTH Aachen University, Medical Faculty, Aachen, Germany

**Keywords:** faculty development, teachers training, competence orientation, teaching competencies, teaching skills, competence development, reflection, CBME, medical education, change management

## Abstract

**Objectives: **Competency-based medical education (CBME) requires factual knowledge to be practically applied together with skills and attitudes. With the National Competence-Based Learning Objectives for Undergraduate Medical Education (NKLM) representing a strong official demand for competence-orientation, it is generally important to explicitly outline its characteristics and review its realisation in teacher trainings. Further requirements are given by the core competencies for medical teachers (KLM). As an example the MQ programme (“Medizindidaktische Qualifikation”) in Baden-Wuerttemberg, a long established and well-accepted training, has been critically revised on this basis, concerning its suitability for the demands of CBME, its needs for adjustment and the efforts to be undertaken for its implementation.

**Methods:** In a systematic quality management process the MQ curriculum and its organisational framing were analysed and further developed in a step-wise comprehensive approach, using the six-step cycle by Kern. The procedures included a thorough needs assessment (e.g. literature research, programme mapping), strategic decisions on structure and content, piloting and evaluation. During the process essential elements of project and change management were considered.

**Results: **The experiences of the MQ example revealed helpful information for key factors to be considered in the pending change process any training provider will be confronted with. Guiding questions were developed related to the process phases. Our analyses showed persistent key points of proven value as stable foundation for change, as well as components needing special consideration to foster competence-oriented aims and transfer into practice: reflection, feedback, application-oriented methods and transparent competence development. These aspects have to be consciously perceived and experienced by participants. Taking this into account, we re-designed the course evidence-based. Besides visualising competencies and their progress, the occasions for reflection and feedback as well as the number of typical, practice-oriented tasks were extended to facilitate self-directed learning, critical self-reflection and individualised solutions. It is shown at what point, in what form and with which purpose these aspects were integrated in the MQ programme. Piloting showed good acceptance by participants, trainers. Preliminary assessment of the outcome is promising.

**Conclusion: **Respecting the high workload, most likely medical teachers will not put CBME concepts into practice without impulses and support. Therefore, in didactical trainings, medical teachers should practice in a competency-based teaching setting and reflect themselves in different professional roles to be able to transfer the experiences to their own educational approach. Trainers and training can serve as models for CBME realisation.

## Zusammenfassung

**Zielsetzung:** Kompetenzbasierte medizinische Ausbildung (CBME) erfordert die praktische Anwendung von Fachwissen in Verbindung mit Fertigkeiten und Haltungen. Die Verabschiedung des Nationalen Kompetenzbasierten Lernzielkatalogs Medizin (NKLM) stellt eine nachdrückliche Aufforderung maßgeblicher Stellen zur Etablierung von Kompetenzorientierung dar. Daher ist es von allgemeinem Interesse, die Charakteristika von Kompetenzorientierung und deren praktische Umsetzung insbesondere in Dozententrainings abzubilden und erfahrbar zu machen. Weitere Anforderungen sind durch die Kernkompetenzen für Lehrende in der Medizin (KLM) gegeben. Auf dieser Basis wurde das MQ Programm („Medizindidaktische Qualifikation“) in Baden-Württemberg, ein langjährig etabliertes und anerkanntes Dozententraining, beispielhaft überprüft und überarbeitet: zum einen hinsichtlich der Passung auf die Anforderungen von CBME und den Veränderungsbedarf innerhalb des Programms und zum anderen hinsichtlich des Aufwandes, der zur Implementierung notwendig ist.

**Methodik: **In einem systematischen Qualitätsmanagementprozess wurden das MQ Curriculum und dessen organisatorische Einbettung analysiert und unter Anwendung von Kerns Six-Step-Zyklus in einem umfassenden Ansatz schrittweise weiterentwickelt. Das Vorgehen beinhaltete eine eingehende Bedarfsanalyse (z.B. Literaturrecherche, Programm-Mapping), strategische Entscheidungen bezüglich Struktur und Inhalt, Pilotierung und Evaluation. Im Verlauf des Prozesses wurden wesentliche Elemente des Projekt- und Changemanagements berücksichtigt.

**Ergebnisse**: Die Erfahrungen des MQ-Beispiels erbrachten hilfreiche Informationen über zu berücksichtigende Schlüsselfaktoren im anstehenden Veränderungsprozess, mit dem jeder Anbieter von Didaktiktrainings konfrontiert sein wird. Dazu wurden Leitfragen zu den jeweiligen Prozessphasen entwickelt. Unsere Analysen zeigten einerseits bewährte und belastbare Eckpunkte als stabile Grundlage für Veränderungsprozesse, andererseits aber auch Komponenten, die besonders überdacht werden mussten, um kompetenzorientierte Ziele und den Transfer in die Praxis zu fördern: insbesondere Reflexion, Feedback, anwendungsorientierte Methoden und transparente Kompetenzentwicklung.

Diese Aspekte müssen von den Teilnehmenden bewusst wahrgenommen und erfahren werden. Auf dieser Basis wurde der Kurs evidenzbasiert umgestaltet. Neben der Visualisierung der Kompetenzen und ihres Aufbaus wurden sowohl die Gelegenheiten für Reflexion und Feedback als auch die Anzahl typischer, praxisorientierter Aufgaben ausgeweitet, um selbstgesteuertes Lernen, kritische Selbstreflexion und individuelle Lösungen zu ermöglichen. Es wird aufgezeigt, an welcher Stelle, in welcher Form und mit welcher Zielsetzung diese Aspekte in das MQ-Kursprogramm eingefügt wurden. Der Pilotlauf zeigte eine gute Akzeptanz durch Teilnehmende und Trainer. Erste Auswertungen der Ergebnisse sind vielversprechend.

**Schlussfolgerung: **In Anbetracht der hohen Arbeitsbelastung werden Lehrende in der Medizin kompetenzorientierte Konzepte höchstwahrscheinlich nicht ohne Impulse und Unterstützung in die Praxis umsetzen. Daher sollten Lehrende in der Medizin in didaktischen Trainings selbst mit kompetenzbasierten Unterrichtssettings konfrontiert sein und sich in verschiedenen professionellen Rollen reflektieren müssen, um die Erfahrungen in ihren eigenen Unterrichtsansatz einfließen lassen zu können. Sowohl die Trainer als auch die didaktischen Trainings können als Vorbilder für die Umsetzung von CBME dienen.

## 1. Introduction

Internationally medical education is turning towards competency-based frameworks and curricular approaches [12], [15], [26], [34] to prepare medical students adequately for the increasing demands on health care and future daily practice [13], [14], [20]. In Germany the National Competence-Based Learning Objectives for Undergraduate Medical Education (NKLM) were recently agreed upon as a framework [11], [18], [http://www.nklm.de/]. Supported by national funding, the MERLIN-group [http://www.merlin-bw.de/], consisting of four medical faculties of Baden-Wuerttemberg, developed competency-based concepts for teaching, learning and assessing, thus gathering experiences in dealing with the NKLM beforehand [27].

The curricular transition towards competency-based medical education (CBME) encounters several challenges and heavily depends on readiness of faculties, as international reports showed [5], [6], [22]. Especially medical teachers are key players in change, being responsible for putting concepts into practice [3], [4], [38]. Acting at the interface between planning and practical implication, teachers have to be familiar with different perspectives on the teaching and learning process. Firstly, they have to understand the aims of competency-based teaching and learning as well as the NKLM role framework, and consider them relevant for professional reality. They need to be aware about the intended change in outcome profile of medical students and in the focus on individual competencies [6], [17], [22], [38]. They should also know and understand the local curricula of the medical faculties in which they are working. Secondly, seen from a teacher perspective, they need to adapt and/or innovate their way of planning, teaching and assessing according to the NKLM and KLM (core competencies for medical teachers) [16], [39], in order to adequately structure and support the process of competence development following constructive alignment [2], [6]. They have to be aware of being role models, not only for their specific medical expertise, but also as medical professionals [31], [38], [40]. In addition, medical teachers need to play an active role in the teaching and learning process, exploring and addressing opportunities and limits. To be able to meet those needs, the critical but constructive reflection of the (changing) circumstances and above all the individual development as a teacher within the system is of key significance. The focus on these abilities becomes even more important since todays’ medical teachers were usually socialised in a different (traditional) way and may therefore tend to teach the way they experienced and are familiar with [4], [5], [6].

These demands and the workload in patient care and research make tailored teacher training programmes essential to support medical teachers [33], [38], [41]. By now the vast majority of German medical faculties offer teachers’ trainings oriented towards defined national standards [28], but in a variety of duration and focus. Most training programmes are being routinely evaluated and adjusted in sense of quality assurance. However, the actual development towards competency-based curricula implies the necessity for fundamental revision and change exceeding usual procedures. 

In Baden-Wuerttemberg, formal training for teaching in medicine (“Medizindidaktische Qualifikation” or MQ) is established since 2001 and has become a standard requirement for habilitation [10], [29], [http://www.medidaktik.de/]. The programme is a two-level curriculum: In the foundation module “MQ I” (120 teaching units) medical teachers acquire basic knowledge of educational principles and methods as well as hands-on skills in relevant formats of medical education. Individual coachings contribute to the transfer into teaching practice. In the advanced module “MQ II” (80 teaching units) specific teaching profiles can be developed (e. g. assessment). The programme has been continuously adapted, reacting to evaluation results and faculty needs. Ideas of CBME were already integrated in trainings.

Based on the NKLM and KLM frameworks the MQ programme has to be critically examined concerning the following questions: To what extent is the MQ programme to professionalize medical teachers (still) suitable for todays’ demands of CBME? Moreover, reflecting the process of analysis and change: What has to be done to adjust a long-term established teachers’ training programme to highlight competence-oriented aspects?

## 2. Methods

The MQ programme was systematically analysed and re-designed in a step-wise comprehensive approach, oriented to the six-step cycle for curriculum development by Kern [25, 33]. Essential elements of project and change management were considered in the process (see Table 1 (Tab. 1)).

### 2.1. Communication

Particular emphasis was laid on early and repeated communication with all parties involved in the programme on all institutional levels. All sorts of informal and formal settings (e.g. meetings, workshops, presentations), and instruments (e.g. focus groups, interviews) were used. The aim of this multi-faceted approach was to clarify interests, align goals, inform about project and create ownership by participating in the process.

#### 2.2. Needs assessment

To determine needs for targeted development further methods were used: a literature analysis, data from a research project on teachers’ professional role perceptions [17], a detailed mapping of the MQ curriculum against core teaching competencies (objectives, content, reached level) [16], [39], a thorough analysis of course evaluations from 2011-2012 (29 courses; 4 medical faculties; written and oral feedback by 383 participants, trainers and administration). The results of these measures were integrated into a SWOT analysis.

#### 2.3. Re-designing of programme

On this basis, the programme was re-designed (e.g. structure, content, methods, materials) and pilot tested in a step-wise approach. All trainers had to participate in 1-2 days trainings. They were introduced to the new concept in detail; they discussed and commented it thus creating a feeling of ownership and stronger commitment.

#### 2.4. Evaluation

Multiple quality management measures were used to evaluate the re-designed programme. At first, the participants were questioned using standardized evaluation forms, structured group discussions as well as informal talks (focus: e.g. content, didactic implementation and extent). Secondly, the trainers involved were interviewed in written and oral form (focus: e.g. content, fit of methods, course structure, organisational topics). Thirdly, the administrative staff was interviewed about feasibility.

## 3. Results

In order to match the upcoming demands of NKLM and KLM, the MQ programme was analysed systematically and comprehensively from both micro and macro perspective with respect to its level of competence orientation (see Table 1 (Tab. 1)). The presentation of results is focused on the basic part of the programme (MQ I) and mainly on conceptual aspects, underpinned additionally by evidence from literature. Initial experiences with the re-worked concept are outlined briefly. The thorough needs assessment revealed

1. persistent key points of proven organisational or content-related value to build upon, as well as
2. components needing adjustments to support competence-oriented aims.

### 3.1. Persistent key points

From an institutional perspective, the MQ programme is well established and embedded in a functioning structure [28], [29]. A comprehensive quality management system is firmly installed on multiple levels to document and ensure programme quality. The programme is accepted and highly valued by all parties involved (e.g. course evaluations WS01/02-WS12/13: n=100 courses; M=1.49±0.17; grade system: 1=best, 5=worst). Its stability offers an ideal environment to experiment and to monitor changes effectively without compromising.

The organisational structure of the MQ programme has proven helpful because of its reliable, systematic design. The standardization of MQ I ensures that all relevant topics and techniques can be addressed in the breadth, promoting competence-orientation. Nevertheless, it offers enough room to focus on individual needs by e.g. microteaching, revision of the own work. In contrast, MQ II provides the flexibility to shape specific teaching profiles. The modules of MQ are organized in blocks of 3-5 days duration, giving the chance of protected time to get an access to the competency-based approach and opportunities to train in practice-oriented tasks [33], [41]. A block concept provides enough flexibility and scope to integrate new elements by replacing or relocating single units. Additionally, working in a relatively stable group creates trust and collegiality, thus fostering individual development by mutual feedback, support and discussion [41], [40]. Concepts and connections are often consolidated in the structured practice phase already highly valued as collegial coaching.

In the overall evaluation it can be concluded that scope and structure of training as well as scientific and political embedding form a solid framework allowing and supporting a shift in emphasis towards more competence-orientation. With regard to content it became clear, that the programme already contained the most relevant topics. The traditional teaching duties (planning, teaching, counseling, assessing, evaluating) and formats (lectures, seminars, bedside teaching) will continue to be relevant in teachers practice.

#### 3.2. Aspects needing adjustment

Even though the MQ programme is founded on the guiding principles of competence-orientation [29][, analyses have shown that it is essential to actively create more awareness and transparency about the acquisition and development of competencies. Specific aspects need to be highlighted and newly arranged to apply the competency-based approach and to make its outcome tangible for the teachers by experience.

##### 3.2.1. Change in perspective

The ability to take on different perspectives plays a key role in further development. Therefore, occasions for guided changes of perspectives were extended, and confrontation was intensified to promote that reflective competence (see Table 2 (Tab. 2)).

1. It is fundamental to be able to switch to a meta-perspective to assess aspects (e.g. methods, circumstances, learning progresses) in a more objective way [1], [31], [38] and to get access to the overall idea of CBME. Acting on the meta-level enables to recognize principles and relations, as well as to anticipate needs and reactions [30], [36]. In the implementation, course elements function as examples; trainers are serving as models by actively stepping aside and focussing on specific aspects (e.g. “Before we continue: How can you make use of this?”).
2. The switch to the meta-level is crucial for self-reflection [32], [31], [37], [36]. Throughout the course participants are often explicitly asked or implicitly inspired to reflect their way of teaching and individual professional development (e.g. self-assessment before and after microteaching sequences and peer coaching sessions: “What did work out, what not and why?”).
3. Another key element needing extensive training is to give feedback [21], [23], [42], [43]. Overall, more practically relevant situations for giving feedback were established, accented and reflected in the group (e.g. bedside teaching unit, peer review of group work results).

##### 3.2.2. Methods and media

Competencies can only become apparent and assessable in practical application [9], [13]. Methods offer ways to create learning opportunities [42].

1. Since entry levels of participants’ skills and external requirements have increased, a unit addressing interactive methods was moved from the advanced module to the basic module to develop the participants’ toolbox. In this unit, a variety of methods for different purposes has to be worked out, later on integrated into course planning and applied in a real teaching setting.
2. A special focus was put on digital media as support for teaching and learning. Web-based media, independent from time and place, can be used to boost self-directed learning and communication [8], [22], [35]. Throughout the course a learning platform was implemented to prepare for courses, to submit pre-and post-assignments. Additionally, chances and risks of digital media application in teaching scenarios (e.g. blended learning formats) are experienced, discussed and reflected.
3. Major attention was paid to systematic implementation of application-oriented methods that combine knowledge, skills and attitudes in context-related tasks of increasing complexity (see Table 3 (Tab. 3)). In sense of constructive alignment, defined tasks were explicitly used for formative (self-)assessment, revealing the actual level of competence. The practice phase itself was expanded as a method to form a temporary climax at the end of each block, applying and documenting e.g. target group analysis, defining competence levels and learning objectives, tabular course design, course conducting, as well as 360°-feedback (self-reflection, peer feedback, student evaluation, expert review).

##### 3.2.3. Transparent structure

Visualisations are effective tools to create transparency.

**Figure 1:**
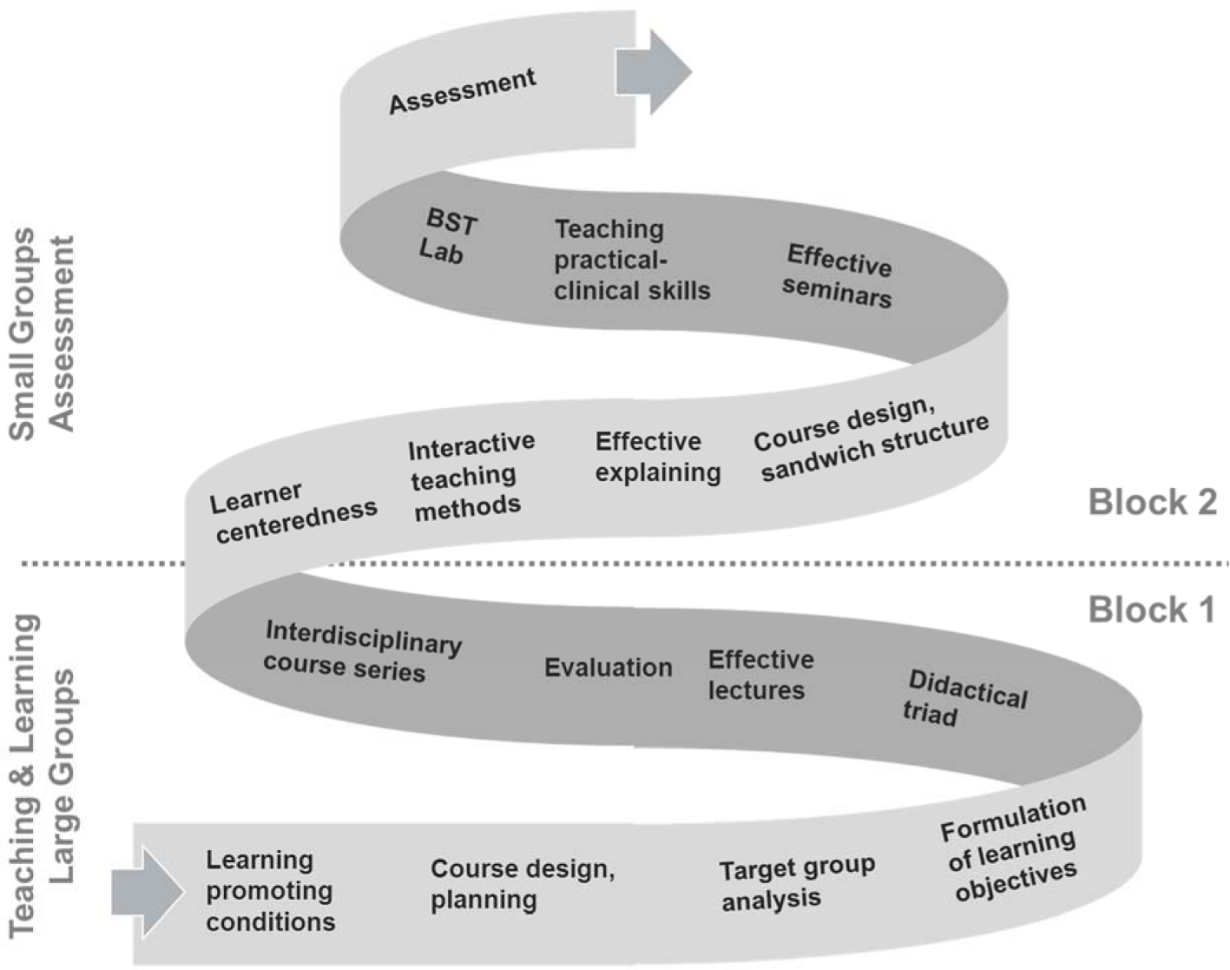
Competence spiral as a structuring element in the foundation module of the MQ programme. MQ I is organized in 2 attendance phases of 3 day blocks, each followed by a practice phase. The spiral indicates the progress in complexity of content and tasks corresponding to an increasing level of competence. The tasks offer opportunities responding to individual levels and needs.

1. The “competence spiral” (see Figure 1 (Fig. 1)) was conceptualized as a structuring element [19], [42]. It illustrates the topics, their chronological sequence and increasing complexity in an overview. This step-wise acquisition of competencies is exemplified in table 3 (Tab. 3). The “competence spiral” is present in every course room as well as referred to in presentations at decisive points as an element of reflection.
2. In addition, icons were designed to display the core competencies of medical teachers in all course materials. As on signposts, pictographs are a form of visual communication with high recognition value, giving orientation and simplified information to the viewer. Combinations of multiple icons could be used to display coherence of several core competencies in one unit. Both tools contribute to create overview and transparency, which provide a basis for increasing awareness and fostering sustainable use.

#### 3.3. Piloting and Evaluation

MQ I was conducted in the re-designed concept, with trainers being introduced to it beforehand (see Table 1 (Tab. 1)). Trainers reported in evaluations that the new concept promoted competence-oriented understanding and skills in participants based on discussions and task results, measuring learning outcome at several stages of the programme. Preliminary results confirm the positive impression (e.g. deeper reflection of collegial coaching). A closer look on outcome results will be given in a separate article. Quantitative and qualitative course evaluations revealed a continuing high quality of implementation for the modified concept (evaluations SS13-WS 14/15: n=19 courses; M=1.46±0.18).

## 4. Discussion

This progress report provides an example for a quality management process concerning a long-time established faculty development programme. A review like this has become inevitable for any teachers training because of the actual requirements of competence-orientation (in NKLM and KLM) that exceed usual evaluations. Following the idea of constructive alignment, teachers’ professionalization programmes have to change simultaneously with or even proactively to the basis and focus of medical education (graduate profile) [5], [24]. Subsequently the results are discussed from a superior point of view, since details of the MQ concept were already explained based on evidences. The main focus is to reflect on possibilities of transferring the results to other faculty development programmes.

An important aspect that has to be taken into account to enable the systematic development of competencies is the organisational structure of measures. Different from our situation, many faculty development programmes consistently make use of shorter courses with various topics at individual choice. Generally, this means changing participants with a heterogeneous level of competencies increasing the challenge for trainers in courses as well as for faculty to predict teachers’ competence profiles. There has to be an overall planned structure attuned to the principles of CBME scaffolding the development of teaching competencies [6], [22], [33]. Every course has to be transparently embedded in this big picture and oriented towards the overall goal. After all, programme directors and trainers alike need to be aware of their mandate and responsibility in preparing medical teachers.

On this basis, it is relevant to consider the key factors reflection and feedback [37], [41], [40]. The awareness of the meta-level, the ability to consciously and regularly switch to a more objective view and the communication of observations are a driving force for competence development. This applies for all three perspectives involved (see Table 2 (Tab. 2)). It is crucial to approach these aspects comprehensively in any faculty development programme.

It is of comparable significance to offer ways to make CBME tangible and individually accessible. Frequent solving of application-oriented tasks empowers learners of different levels of competence to link theoretical knowledge to professional practice and generates the ability for target-oriented action. In particular, digital media open up new opportunities in this context [7], [8], [22], [35]. Contents, materials and tasks can be relocated before and/or after courses. Thus room for important aspects requiring attendance time as well as opportunities and impulses for self-directed learning can be created. On the one hand this is a time-efficient proceeding for every party involved, on the other hand, it enables to build upon and react to individual learning progress. Since there are (still) barriers to implement digital media in regular teaching, medical teachers should experience its strength, opportunities and risks in teacher trainings to increase the chance of digital media use in medical education practice. The collegial coaching as a method in the practice phase has gained in impact in two ways: transfer of new concepts into practice and dissemination into the departments.

By defining the core competencies (KLM), the so far implicit requirement profile for medical teachers has become nationally explicit. Transparency plays a major role while implementing CBME, especially on occasions that are focused on matters of teaching. It is easier for teachers to grade their individual status and to follow their progress with the help of defined competencies as points of reference. Visualisations (e.g. icons, competence spiral) and regular addressing of actual status and targeted level of competencies offer simple methods to support this process. In summary, efforts should be intensified to identify, display and reflect the core competencies and their development in teacher trainings.

The quality management shows considerable restrictions. Firstly, it is a long process to generate a shared culture of teaching and learning. As long as not all medical teachers are trained and have internalised the new concepts, students and faculty are confronted with a mixed culture. Efforts are necessary to reach a critical mass of supportive faculty. Critical analysis and restructuring of faculty development programmes have to be seen as an extensive but rewarding investment to initiate and accelerate culture change.

Secondly, trainers (as medical teachers themselves) are responsible for concept realisation. The quality of implementation is difficult to ensure and may vary (e.g. honorary staff, non-specialist staff, staff turnover, untrained staff) even though different measures to uphold quality are in use (e.g. targeted selection, training, exchange meetings, information material, coaching, evaluation and feedback). Programme directors have to continuously monitor and motivate their trainers and trust them after all, because the intended mindset cannot be guaranteed.

Thirdly, competence development is mainly depending on the learners themselves. Participants attend courses with different individual values, attitudes and expectations towards teaching and learning, depending on experiences and teaching reality. Besides, obligation of training (e.g. for habilitation) may collide with individual conceptions. In consequence, this may hinder acceptance, internalisation and/or application of elements of CBME. All the more the stimulation of reflection is required. Last but not least, participants need supervisors’ approval, opportunities and adequate framing to implement new concepts in their teaching practice. At that time, these factors are often not given or insufficient when required.

## 5. Conclusion

Faculty development programmes are essential to disseminate CBME. Especially programme directors and managers need to be continuously aware of and reflect their key role in stimulating the change process. Under time pressure medical teachers will not put CBME concepts into practice without impulses and support. Additionally, the risk of resistance to change and a growing hidden curriculum may be reduced by suitable faculty development measures. All in all, programme adaptation is a rewarding investment in teaching culture for any institution.

## Acknowledgements

We would like to thank Christine Baatz and Sarah Durante, both Tuebingen, for their supportive contribution in the acquisition of data in the early project phase. We highly appreciate the helpful feedback from both of them, as well as from all trainers.

## Competing interests

The authors declare that they have no competing interests. 

## Figures and Tables

**Table 1:**
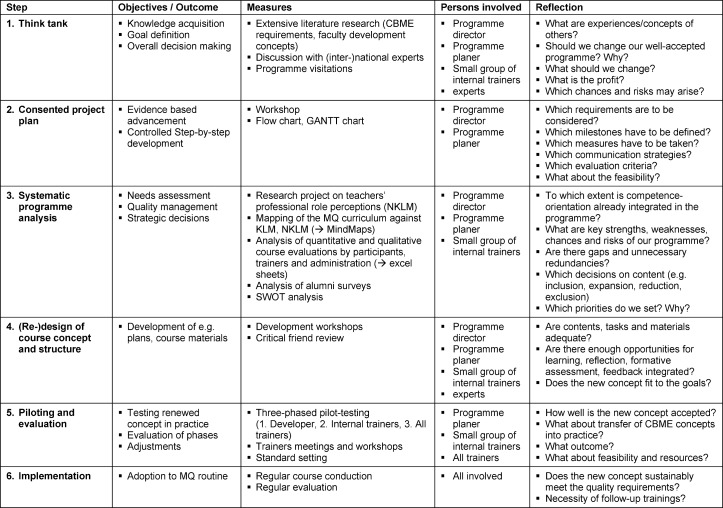
Overview of the change process in the MQ programme

**Table 2:**
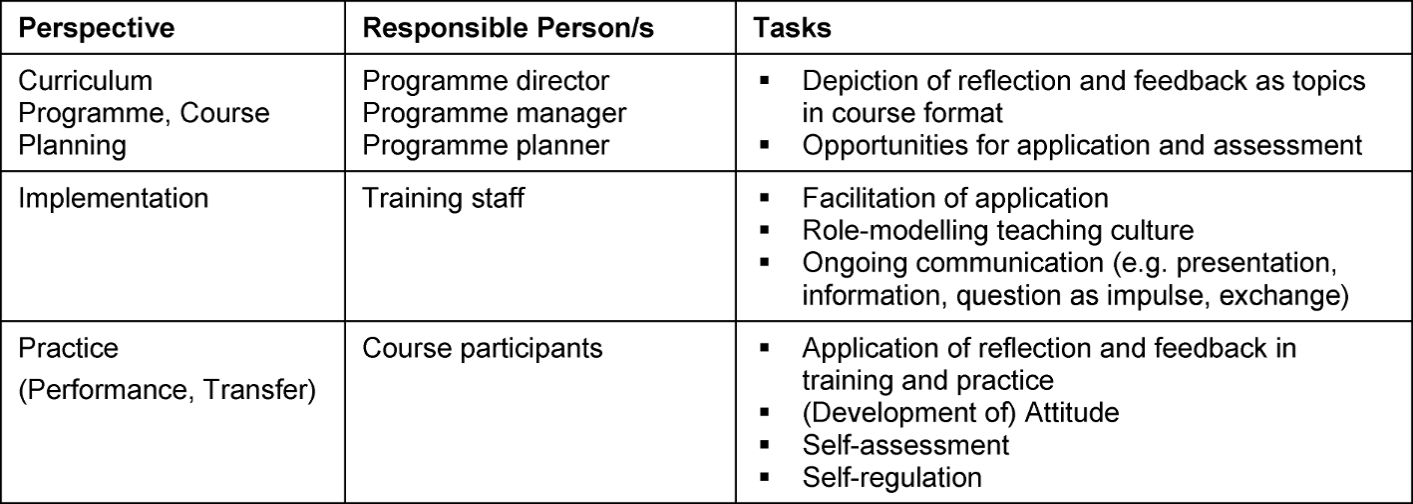
Responsibilities in integrating reflection and feedback as key factors for competence development in teacher trainings/practice

**Table 3:**
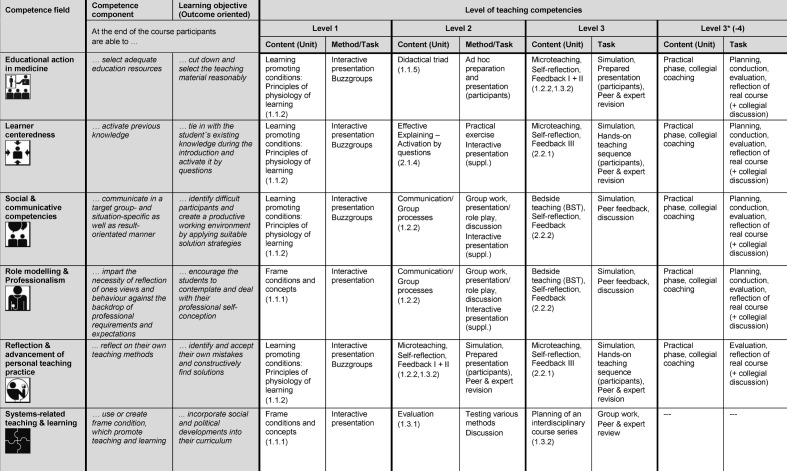
Exemplified development in teachers’ core competence fields during MQ I. For every core competence a competence component and a learning objective were selected as an example. The development of this specific objective is depicted on its increasing levels throughout the course with exemplified units and tasks (cp. figure 1). The levels of competencies were defined as; 1=knowledge/reproduction, 2=understanding, 3=demonstration in practice (during course), 3*=demonstration in real teaching setting, 4=integration in routine. The units in brackets are named in the following form: block, day, unit (e.g. block 1, day 1, unit 1 → 1.1.1). Topics of MQ I are subsequently deepened at individual choice in the advanced module MQ II.
